# 3D conductive coupling for efficient generation of prominent Fano resonances in metamaterials

**DOI:** 10.1038/srep27817

**Published:** 2016-06-14

**Authors:** Zhiguang Liu, Zhe Liu, Jiafang Li, Wuxia Li, Junjie Li, Changzhi Gu, Zhi-Yuan Li

**Affiliations:** 1 Institute of Physics, Chinese Academy of Sciences, Beijing 100190, China; 2 Collaborative Innovation Center of Quantum Matter, Beijing, 200092, China

## Abstract

We demonstrate a 3D conductive coupling mechanism for the efficient generation of prominent and robust Fano resonances in 3D metamaterials (MMs) formed by integrating vertical U-shape split-ring resonators (SRRs) or vertical rectangular plates along a planar metallic hole array with extraordinary optical transmission (EOT). In such a configuration, intensified vertical E-field is induced along the metallic holes and naturally excites the electric resonances of the vertical structures, which form non-radiative “dark” modes. These 3D conductive “dark” modes strongly interfere with the “bright” resonance mode of the EOT structure, generating significant Fano resonances with both prominent destructive and constructive interferences. The demonstrated 3D conductive coupling mechanism is highly universal in that both 3D MMs with vertical SRRs and vertical plates exhibit the same prominent Fano resonances despite their dramatic structural difference, which is conceptually different from conventional capacitive and inductive coupling mechanisms that degraded drastically upon small structural deviations.

Plasmonic Fano resonances in nanostructures and metamaterials (MMs) have attracted great attention due to their promising applications in ultrasensitive biosensing, high-contrast optical imaging, high-quality optical waveguides, as well as new types of nonlinear and switchable metamaterials^1^,^2^,^3^,^4^. As a result, the efficient generation of plasmonic Fano resonances has raised extensive interest in recent years, and a wide variety of approaches and geometries have been proposed and explored, such as nanoparticle clusters^5^,^6^, nonconcentric nanoparticles^7^,^8^, composite split-ring resonators (SRR)^4^,^9^,^10^, perforated metallic films^11^, metallic photonic crystals^12^, etc. Generally, two schemes could be utilized for the generation of Fano resonances. The first type of plasmonic Fano resonance could be induced by the coherent interference between spectrally-overlapped superradiant dipole modes and subradiant high-order modes, i.e. by the coupling between broad bright modes and narrow dark modes^8^,^13^,^14^. For example, in non-concentric ring/disk cavity systems, the broad dipole modes of the disks were interfered with the narrow high-order modes (dark modes) of the rings, resulting in Fano resonance with strong sensitivity to the environments^8^,^10^. As the second type, the plasmonic hybridization between spectrally-overlapped dipole modes (both are bright modes) of adjacent nanoparticles could also generate versatile Fano resonances, which has been widely employed in the so-called plasmonic oligomers (or nanoparticle clusters)^15^,^16^,^17^. So far, most plasmonic Fano resonances were reported in two-dimensional (2D) systems and more or less encountered constraints: in the former case, the high-order dark resonance modes were normally weak and not easily accessible^3^, for the latter type, the plasmonic hybridizations were extremely sensitive to the plasmonic gaps between neighboring nanoparticles, which are difficult for controllable fabrications^15^,^18^.

The coupling mechanisms available for the plasmonic Fano resonances could be summarized into three types, i.e. capacitive (electric) coupling, inductive (magnetic) coupling and conductive (connective) coupling^18^,^19^,^20^. Among them, the capacitive coupling depends on the charge oscillations on the two sides of the plasmonic gaps^15^,^21^ and the inductive coupling relies on the current flows in coil-type configurations^10^,^20^,^22^, both of which are well known and have been widely adopted in the generation of plasmonic Fano resonance^1^,^2^,^3^. In comparison, the conductive coupling has been largely overlooked due to its simple configuration at the first sight (i.e. the 2D conductive structures look like simple electric connection of individual elements), although it has exhibited strong impacts on the modifications of plasmonic properties in systems such as gold nanorod dimers^23^,^24^, theta-shaped ring-rod nanostructures^19^, and THz connected SRR structures^18^. The main reason is that the 2D connected elements were generally treated as single conductive structures and the resulted couplings were traditionally rationalized as the interference between the bright dipole modes and the dark high-order modes^19^.

Recently we observed significant Fano resonances from a “nanograter”-like 3D MM formed by integrating vertical U-shape SRRs along a planar metallic hole array and explained the observations phenomenologically and qualitatively by traditional plasmonic hybridizations^25^. In this work we demonstrate an even simpler 3D MMs with micro-plates to generate significant Fano resonance without the use of SRR-type building blocks. It is found that a 3D conductive coupling mechanism is the actual physical mechanism for the efficient generation of prominent Fano resonances in both 3D MMs. In this mechanism, when the metallic hole array with extraordinary optical transmission (EOT) is illuminated by normal incident light, the excited surface plasmon polaritons (SPPs) with electric-field (E-field) polarized perpendicular to the 2D array plane can efficiently activate vertical electric currents in the vertical nanostructures (vertical SRRs or vertical micro-plates), which naturally introduce “dark” modes. This 3D conductive “dark” mode strongly interferes with the “bright” dipole-like resonance mode of the EOT structure, which forms pronounced Fano resonances with both prominent destructive and constructive interferences. We show that once the conditions for this mechanism are satisfied, the prominent Fano resonances could be well preserved no matter the vertical structures are SRRs or micro-plates. Therefore, our 3D conductive coupling induced prominent Fano resonances are very robust and conceptually different from conventional capacitive and inductive coupling schemes that degrade drastically upon small structural deviations.

## Results

### Experimental observations

**Figure 1:**
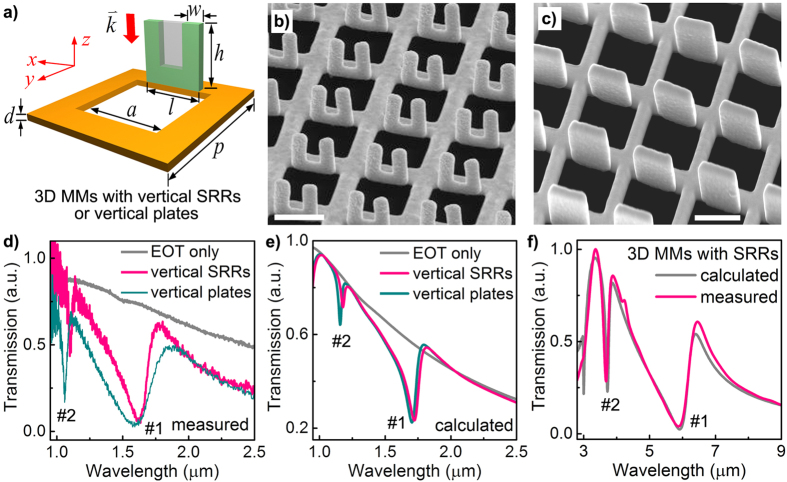
Prominent Fano resonances in 3D MMs. **(a)** Schematic of the unit cell of a 3D MM in which the vertical SRR or vertical plate (SRR filled with the grey area) is conductively connected to one edge of a metallic hole. The free-standing structure is completely composed of Au without any substrate. (**b,c**) Side-view SEM images of the fabricated 3D MMs with (**a**) vertical SRRs and (**b**) vertical plates, respectively. Scale bars: 500 nm. **(d)** As-measured and **(e)** calculated transmission spectra of an EOT structure without any vertical structure and two 3D MMs with different vertical structures under y-polarized light excitation. The Fano resonances of the two 3D MMs are well preserved under the dramatic structural changes. Structural parameters: *p* = 750 nm, *a* = 560 nm, *d* = 50 nm; *l* = 430 nm, *h* = 560 nm, *w* = 150 nm; width and height of the vertical plates: *l*_*p*_ = 430 nm, *h*_*p*_ = 560 nm. **(f)** Measured and calculated transmission spectra of a 3D MM under y-polarized light excitation in the mid-infrared wavelength region, agreeing very well with each other. Structural parameters: *p* = 3 μm, *a* = 2μm, *d* = 80 nm, *l* = 1 μm, *h* = 1.45 μm, *w* = 0.25 μm.

Figure 1a illustrates the schematic of the 3D MMs in our studies, which are formed by vertical U-shape SRRs or vertical rectangular micro-plates standing along a planar metallic hole array. The U-shape SRRs have been the popular and widely accepted building blocks for various 2D and 3D MMs in which the electric resonances could be excited by E-field parallel to the two arms (*E*_*z*_) and the magnetic resonances can be excited by E-field perpendicular to the two arms while parallel to the “U-plane” (*E*_*x*_)^26^. It should be mentioned that in most cases, the SRR-based MMs employed isolated SRRs with planar^27^, vertical^28^,^29^ or multi-layer arrangements^30^, which mainly relied on electric coupling, magnetic coupling or their combinations. In comparisons, the configurations like in Fig. 1a are not traditionally considered due to the lack of knowledge in conductive coupling, as well as the difficulties in fabrications of such 3D nanostructures.

By using an *in situ* focused-ion-beam (FIB) irradiation-induced folding technique that we recently developed (see Methods)^25^, it is very sophisticated to realize such kinds of 3D MMs in free-standing Au films, as shown in Fig. 1b (a 3D MM with vertical SRRs) and Fig. 1c (a 3D MM with vertical plates), respectively. Surprisingly, the transmission spectra of both 3D MMs exhibit significant Fano-like resonances in both long wavelength and short wavelength regions (named as Fano resonance #1 and #2, respectively) as plotted in Fig. 1d, forming sharp contrast to the spectrum of the EOT structure without any vertical structures (grey line in Fig. 1d). This preservation of Fano resonances upon drastic structural changes is well verified by our numerical simulations in Fig. 1e, indicating that the central gap of the vertical SRRs does little effects to the generation of prominent Fano resonances. These findings are very important from two aspects. Firstly, compared to the 3D MMs with SRRs, the 3D MMs with micro-plates have obvious advantages on fabrication efficiency (33% reduction in fabrication time) due to their simpler geometry and mechanical stabilities due to the less overhanging structures. Secondly, these observations indicate an underlying physical mechanism responsible for the generation of prominent Fano resonances, which might be universal for the designs of other types of 3D MMs with versatile Fano resonances and thus deserves in-depth studies.

### Analysis and modellings

One may notice that except the emerging of both Fano resonances in similar wavelength regions with the same trend, some details of the measured and calculated results in Fig. 1d,e are not well matched. In comparison, the measured transmission spectrum of a 3D MM with SRRs in mid-infrared wavelength region, as plotted in Fig. 1f, is nearly perfectly matched with the calculation. This could be caused by the fact that in the realistic cases, the structures with tiny features normally suffer from relative larger fabrication imperfections than structures with larger sizes. Meanwhile, the material constants employed in modellings may deviate from realistic materials more seriously in high-frequency region than those in low-frequency region. Therefore, to effectively find out the underlying physical mechanism responsible for the Fano resonances, we move the studies to mid-infrared wavelength region, where experimental observations and numerical simulations are more consistent (Fig. 1f) and the principles can be generally applied.

**Figure 2:**
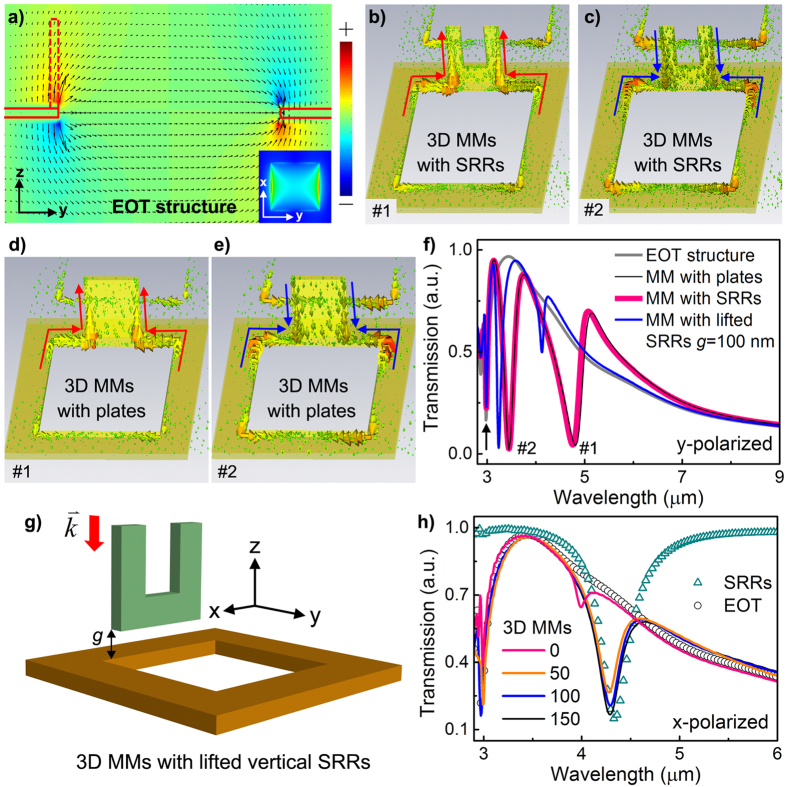
Modellings and simulations. **(a)** Simulated E-field distribution (indicated by dark arrows) of an EOT structure (a pure metallic hole array without SRRs) in the yz plane. *E*_*z*_ is plotted in color. The solid red lines outline the structural profile and the dashed red lines indicate the position of the vertical structure where *E*_*z*_ is naturally intensified and can efficiently drive the electric current flow in the vertical structure. (Inset) Dipole-like E-field distribution of EOT structure in xy plane. **(b–e)** Simulated current distributions of the 3D MMs with **(b,c)** vertical SRRs and **(d,e)** vertical plates, respectively, at two distinct modes (named as #1 and #2). As noted by the arrows, Mode #1 represents an in-phase conductive current flow while Mode #2 denotes an anti-phase current flow. The similarities between **(b,d)**, as well as **(c,e)**, indicate the same underlying physical mechanism. **(f)** Simulated transmission spectra of an EOT structure, a 3D MM with vertical plates, a 3D MM with vertical SRRs and a 3D MM with lifted vertical SRRs with a gap distance of *g* under y-polarized light excitation. The arrow indicates the periodicity of the structural lattice. **(g)** Schematic of the unit cell of a 3D MM in which the vertical SRR is lifted above the EOT structure by a gap distance of *g*. **(h)** Simulated transmission spectra of an EOT structure, an SRR array, and 3D MMs with different *g* (unit: nm) under x-polarized excitation. Structural parameters: *p* = 3 μm, *a* = 2 μm, *d* = 80 nm, *l* = 0.9 μm, *h* = 0.8 μm, *w* = 0.25 μm; width and height of the vertical plates: *l*_*p*_ = 0.9 μm, *h*_*p*_ = 0.8 μm.

On the other hand, since reported in 1998 by Ebbesen and co-workers^31^, the EOT in a metallic hole array has been well recognized as the result from two resonances, i.e. the surface plasmon resonances (SPRs) due to the periodicity and the localized plasmonic waveguide modes within the holes^32^,^33^. Importantly, the SPRs of the EOT structure could induce significantly enhanced E-field along the metallic holes in the z direction (*E*_*z*_) when excited by y-polarized light, as plotted in Fig. 2a. Therefore, when a vertical SRR or vertical plate is placed within the intensified *E*_*z*_ field (as the dashed outlines in Fig. 2a), the electric resonances of the vertical structure could be naturally and efficiently excited. The corresponding excitation efficiency will be the highest if the vertical structure is electrically connected to the edge of the metallic hole due to the fact that the intensified *E*_*z*_ field is strongest at the air/metal interface. As a result, Fig. 2b–e plot two resonant electric current flows along the vertical SRRs and vertical plates of the 3D MMs, respectively. It can be seen that Mode #1 represents an in-phase conductive current flow while Mode #2 denotes an anti-phase current flow. Due to symmetry constraints, only currents that are parallel to the incident E-field can radiate into the far field^18^. Therefore, under y-polarized incident E-field, both modes (Modes #1 and #2) associated with vertical (z-direction) currents flows are non-radiative and naturally “dark” modes. These 3D conductive connection induced dark modes could strongly couple with the dipole-like SPR resonance of the EOT structure (the whole process is called as 3D conductive coupling), which finally results in prominent Fano-like resonances. As plotted in Fig. 2f, the sharp and asymmetric spectral profiles of the Fano resonance (red and black lines) in the 3D conductive MMs exhibit dramatic differences to that of the EOT structure (grey line). More importantly, it can be seen that the transmission spectra from the 3D MMs with vertical SRRs (red line) and vertical plates (black line) are almost the same, mainly caused by the fact that the 3D conductive coupling processes in both structures are mostly similar, as illustrated in Fig. 2b,e.

To further demonstrate the importance of the 3D conductive coupling mechanism, the vertical SRRs are intentionally lifted from the in-plane EOT structure in Fig. 2g, in the case of which capacitive coupling could be initiated when the gap distance (*g*) is larger than zero. It can be seen from Fig. 2f that when the gap distance is increased to 100 nm (corresponding to ~λ/45), i.e. when the condition changes from conductive coupling to capacitive coupling, the peak-to-dip depth of Fano resonance #1 degrades dramatically (blue curve). This observation clearly proves that the 3D conductive coupling mechanism plays a major role in the generation of prominent Fano resonance #1. It should be emphasized that the 3D conductive coupling induced Fano resonances only exist under y-polarized excitation. When excited by x-polarized incident light, as shown in Fig. 2h, the transmission of the 3D MMs with different gap distance shows little spectral shift. In this case, the spectra of the 3D MMs simply represent the linear spectral overlapping between the SRR and EOT structures, indicating there is almost no couplings between the SRR and EOT structures when *g* > 50 nm although the magnetic resonance of the SRR (plotted by the triangles in Fig. 2h) could be separately excited by x-polarized light. It should be mentioned that the tiny spectral dip at 4 μm in the case of *g* = 0 results from the modified magnetic resonance of the SRR when it is connected to the EOT structure.

**Figure 3:**
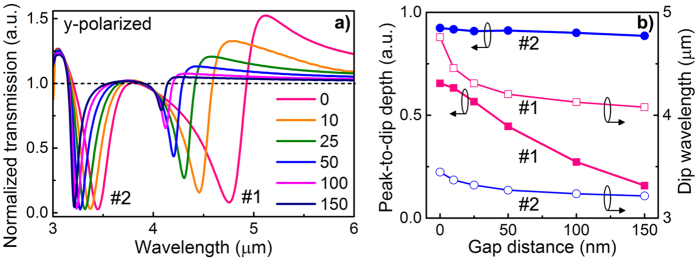
Comparisons between 3D conductive coupling and capacitive coupling. **(a)** Normalized transmission spectra of the 3D MMs with SRRs to that of EOT structure under different gap distances (unit: nm) upon y-polarized excitation. **(b)** (left) Absolute peak-to-dip depth and (right) dip wavelength as a function of the gap distance for resonances #1 and #2, respectively. Structural parameters are the same as in Fig. 2 except the gap distance *g*. Fano resonance **#**1 is mainly resulted from the 3D conductive coupling, which will be more discussed in following studies.

For quantitative comparisons, in Fig. 3a we normalize the transmission spectra of the 3D MM to that of the EOT structure under y-polarized excitation when the coupling scheme varies gradually from 3D conductive coupling (*g* = 0 nm) towards capacitive coupling (*g* > 0 nm). One can clearly see that for *g* = 0 nm and near Fano resonance #1, significant constructive (enhanced by ~52% at the peak) and destructive (suppressed by ~92% at the dip) interference effects are observed in the case of y-polarized excitation. This is quite consistent with the classical understanding that Fano resonance describes both destructive and constructive interferences between a discrete state and a continuum state^2^. Meanwhile, it is found that for Fano resonance #1, both the resonance wavelength and the amplitude of constructive and destructive interferences are highly sensitive to the gap distance. Its peak-to-dip depth drops quickly by 75% when the 3D MM deviates slightly from conductive coupling (*g* = 0 nm) to capacitive coupling with a gap distance as small as λ/20 (*g* = 150 nm), as shown in Fig. 3b, further illustrating the critical role of the 3D conductive coupling. In comparison, both resonance wavelength and amplitude of Fano resonance #2 change more slowly with the gap distance. This is because for the anti-phase coupling case of Mode #2, the repelling currents in SRR and EOT structure are relatively independent even when the two structures are electrically conducted. Therefore, the 3D conductive coupling effect in Fano resonance #2 is much weaker than that in Fano resonance #1.

From above analysis, it is now clear that the prerequisites for 3D conductive coupling include two parts: the first part is a planar structure that couples efficiently with incident light and supports SPR-enhanced vertical E-field (*E*_*z*_); the second part is a vertical structure that can sustain vertical electric resonances within the intensified *E*_*z*_ field. As long as these two prerequisites are satisfied, prominent Fano resonances could be generated without the need of complicated formulas, models, or mechanisms^34^. Therefore, since the electric current flows are mostly along the outer edges of the SRRs under y-polarized excitation (Fig. 2b,c), simply replacing the SRR structure with a vertical rectangular plate of the same size does not change the two prerequisites for prominent Fano resonances (Fig. 2d). Consequently, the 3D MMs with vertical SRRs and vertical plates possess significant Fano resonances at nearly the same spectral positions, as shown in Fig. 1d–f, verifying the robust and universal generation of Fano resonances with the 3D conductive coupling mechanism. One may notice that the position and depth of the measured Fano resonance #1 in Fig. 1d are nearly unchanged while its width is broadened due to the less confinement of the electric charges, which induces extra non-radiative losses. Nevertheless, this preservation of Fano resonances when deviating dramatically from vertical SRRs to vertical plates clearly proves the robustness upon fabrications, which are highly desirable for large-scale or stream-lined fabrications. In other words, the introduction of Fano resonances in our 3D MMs is simply dependent on the effective conductive coupling length. Therefore, by engineering the 3D conductive coupling length through varying the size of the metallic holes, the width and height of the vertical structures, the Fano resonances in 3D MMs could be widely tuned from near-infrared to mid-infrared wavelength region, as illustrated in Fig. 1d,f, which are very preferable for applications in tunable MMs.

### Polarization-independent features of Fano resonances

**Figure 4:**
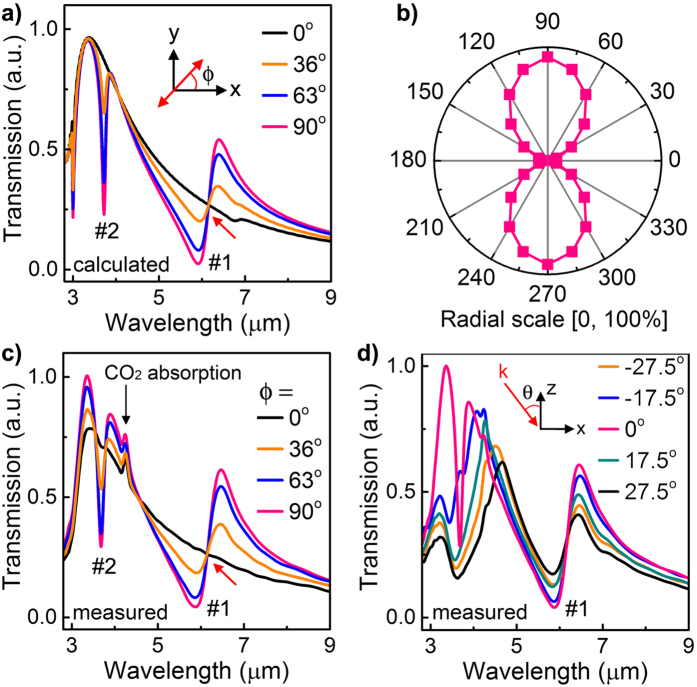
Prominent Fano resonances with polarization-independent spectral position. **(a)** Calculated and **(c)** measured transmission spectra of a 3D MM under normal incidence with different polarization angle *ϕ* as illustrated in the inset. *ϕ* = 90° means y-polarized excitation. The small peaks at approximately 4.2 μm are caused by the unstable absorption from CO_2_ in the FTIR chamber. **(b)** Polar plot of the calculated suppression factor (defined as the difference in transmission between the 3D MM and EOT structure divided by the transmission of the EOT structure) at the dip wavelength of Fano resonance **#**1 as a function of the polarization angle under normal incidence. **(d)** Measured transmission spectra of the 3D MM under y-polarized excitation with different incident angle *θ* as illustrated in the inset. Structural parameters are the same as in Fig. 1f. The variations of dip wavelength of Fano resonance #1 in (**a–d**) are less than 2%, while the resonance strengths are changed due to the different excitation efficiency of SPPs under varied illumination schemes.

Another important feature of the proposed 3D conductive coupling is that the induced Fano resonance #1 exhibits characteristics of localized resonance. As the simulation results shown in Fig. 4a, the position of Fano resonance is well preserved under normal incidence, irrespective of the polarization angles (except that both Fano resonances disappear under x-polarized excitation). The suppression factor at the Fano resonance (#1) dip versus the in-plane polarization angle exhibits a strong dipole-like pattern in the polar plot (as shown in Fig. 4b), indicating the strongly localized and anisotropic features of the Fano resonance. Interestingly, there is a crossing point near Fano resonance #1 where the transmission is fixed for all polarization angles (as indicated by the red arrow Fig. 4a), which is highly preferable for polarization-insensitive optical sensing. These features, including the crossing point, are well verified by our experimental results in Fig. 4c. Moreover, measurements under y-polarized excitation (Fig. 4d) also show that the position of Fano resonance #1 is almost independent on the illumination angle under y-polarized excitation, while Fano resonance #2 that is less affected by the 3D conductive coupling changes dramatically. This robustness of Fano resonance #1 (with wavelength variation of less than 2% for both simulations and measurements in Fig. 4a–d) results from the facts that except the excitation efficiency, the different illumination schemes in Fig. 4a–d do not change the basic conditions for the generation of Fano resonances, i.e. the E-field of the planar SPPs is intrinsically along the vertical direction and the SRRs are vertically connected with the planar structures. Therefore, it could be concluded that the 3D conductive coupling induced Fano resonance #1 is a type of localized resonance and thus its position is polarization-insensitive. These interesting polarization properties of the 3D conductive coupling induced Fano resonances may provide new prospects for the emerging areas of meta-surfaces^35^,^36^.

## Discussions and Conclusions

In summary, we have demonstrated a 3D conductive coupling mechanism for the efficient generation of robust and prominent Fano resonances in a new type of 3D MMs. Based on the 3D conductive coupling mechanism, only two simple prerequisites are required for the generation of Fano resonances, i.e. a planar structure providing intensified vertical E-field on the surface (like EOT structure) and a vertical structure sustaining electric resonances (like vertical SRRs or vertical plates). In such a configuration, electric “dark” modes could be naturally induced and strongly interfere with the “bright” dipole-like resonance mode of the EOT structure. As a result, we have observed pronounced Fano resonances with both prominent destructive and constructive interferences in both simulations and experiments. Moreover, we have shown that the 3D conductive coupling mechanism is highly universal in that both 3D MMs with vertical SRRs and vertical plates exhibited the same prominent Fano resonances although their structural geometries were largely different. Last but not least, the 3D conductive coupling induced Fano resonance exhibits characteristics of localized resonance, of which the wavelength position is immune against certain changes in illumination polarizations. Therefore, our 3D conductive coupling mechanism is simple, universal, robust, and conceptually different from conventional capacitive and inductive coupling mechanisms that degraded drastically upon small structural deviations. This work could provide a new methodology for the efficient generation of Fano resonances with simple geometries, fast fabrications, stable mechanical strength, etc., holding potential applications in enhanced extraordinary optical transmission, color displays, optical sensing, etc., as well as opening up new prospects for the emerging areas of metasurfaces^35^,^36^.

## Methods

### Sample fabrications

The 3D MMs were fabricated with a focused-ion-beam (FIB) irradiation-induced folding technique on self-supporting Au films^25^. Specifically, Si substrates were cleaned and spin-coated with a 1.2 μm layer of S1813 photoresist, then baked at 115 °C for 2 min and deposited an 80-nm-thick Au film using magnetron sputtering. To get suspended Au film, the sample was immersed in acetone for 24 h to fully dissolve the resist and a 100 μm × 100 μm Cu grid was dipped into the solution to pick the film up, which was then dried by N_2_ in ultra-clean room to get a flat and self-supporting Au film. Pre-designed patterns were automatically cut by FIB system (FEI helios 600i). Subsequently, the FIB spot was continuously scanned along the bottom edge of the patterned structures, which folded the structures naturally by the ion-implantation induced stress. Through properly controlling the ion dose, a maximum folding angle of 90° could be realized. The acceleration voltage of Ga^+^ was 30 kV. An ion-beam current of 40 pA was used for the structure processing.

### Optical characterizations

Transmission spectra were collected by using a × 15, 0.4 numerical aperture reflective objective lens on an optical microscope (Hyperion 2000) and coupled to a Fourier-transform infrared spectrometer (Vertex 80, Bruker) through a 40 μm × 40 μm spatial aperture. A homemade aperture was inserted after the reflective objective to confine the illumination cone with a conical angle of ~5°, and the samples were tilted correspondingly to obtain normal incidence during measurement. The transmission spectra were calibrated using air (holes of the grid) as a reference.

### Numerical simulations

The transmission spectra and E-field distributions of the structures were simulated by using the finite-difference time-domain (FDTD) method. The E-field monitors were placed 5 nm above the metal surface. The current distributions were calculated by the commercial software package CST Microwave Studio based on the finite integration method. We used realistic parameters describing gold’s lossy properties, with an electric conductivity of 4.561 × 10^7^ Sm^−1^.The surface current distributions were obtained using an H-field/surface current monitor.

## Additional Information

**How to cite this article**: Liu, Z. *et al*. 3D conductive coupling for efficient generation of prominent Fano resonances in metamaterials. *Sci. Rep*. **6**, 27817; doi: 10.1038/srep27817 (2016).

## References

[b1] Luk’yanchuk B. . The Fano resonance in plasmonic nanostructures and metamaterials. Nat. Mater. 9, 707–715 (2010). 20733610 10.1038/nmat2810

[b2] Miroshnichenko A. E., Flach S. & Kivshar Y. S. Fano resonances in nanoscale structures. Rev. Mod. Phys. 82, 2257–2298 (2010).

[b3] Rahmani M., Luk’yanchuk B. & Hong M. H. Fano resonance in novel plasmonic nanostructures. Laser Photonics Rev. 7, 329–349 (2013).

[b4] Wu C. H. . Fano-resonant asymmetric metamaterials for ultrasensitive spectroscopy and identification of molecular monolayers. Nat. Mater. 11, 69–75 (2012). 22081082 10.1038/nmat3161

[b5] Mirin N. A., Bao K. & Nordlander P. Fano Resonances in Plasmonic Nanoparticle Aggregates. J. Phys. Chem. A 113, 4028–4034 (2009). 19371111 10.1021/jp810411q

[b6] Fan J. A. . Fano-like Interference in Self-Assembled Plasmonic Quadrumer Clusters. Nano Lett. 10, 4680–4685 (2010). 20923179 10.1021/nl1029732

[b7] Hao F. . Symmetry Breaking in Plasmonic Nanocavities: Subradiant LSPR Sensing and a Tunable Fano Resonance. Nano Lett. 8, 3983–3988 (2008). 18831572 10.1021/nl802509r

[b8] Sonnefraud Y. . Experimental Realization of Subradiant, Superradiant, and Fano Resonances in Ring/Disk Plasmonic Nanocavities. Acs Nano 4, 1664–1670 (2010). 20155967 10.1021/nn901580r

[b9] Pryce I. M., Aydin K., Kelaita Y. A., Briggs R. M. & Atwater H. A. Highly Strained Compliant Optical Metamaterials with Large Frequency Tunability. Nano Lett. 10, 4222–4227 (2010). 20857941 10.1021/nl102684x

[b10] Zhang Q. . Multiple Magnetic Mode-Based Fano Resonance in Split-Ring Resonator/Disk Nanocavities. Acs Nano 7, 11071–11078 (2013). 24215162 10.1021/nn4047716

[b11] Garcia-Vidal F. J., Martin-Moreno L., Ebbesen T. W. & Kuipers L. Light passing through subwavelength apertures. Rev. Mod. Phys. 82, 729–787 (2010).

[b12] Christ A., Tikhodeev S. G., Gippius N. A., Kuhl J. & Giessen H. Waveguide-plasmon polaritons: Strong coupling of photonic and electronic resonances in a metallic photonic crystal slab. Phys. Rev. Lett. 91, 183901 (2003). 14611284 10.1103/PhysRevLett.91.183901

[b13] Hao F., Nordlander P., Sonnefraud Y., Van Dorpe P. & Maier S. A. Tunability of Subradiant Dipolar and Fano-Type Plasmon Resonances in Metallic Ring/Disk Cavities: Implications for Nanoscale Optical Sensing. Acs Nano 3, 643–652 (2009). 19309172 10.1021/nn900012r

[b14] Dregely D., Hentschel M. & Giessen H. Excitation and Tuning of Higher-Order Fano Resonances in Plasmonic Oligomer Clusters. Acs Nano 5, 8202–8211 (2011). 21879759 10.1021/nn202876k

[b15] Hentschel M. . Transition from Isolated to Collective Modes in Plasmonic Oligomers. Nano Lett. 10, 2721–2726 (2010). 20586409 10.1021/nl101938p

[b16] Bao K., Mirin N. A. & Nordlander P. Fano resonances in planar silver nanosphere clusters. Appl. Phys. A-Mater. 100, 333–339 (2010).

[b17] Hentschel M., Dregely D., Vogelgesang R., Giessen H. & Liu N. Plasmonic Oligomers: The Role of Individual Particles in Collective Behavior. Acs Nano 5, 2042–2050 (2011). 21344858 10.1021/nn103172t

[b18] Al-Naib I. . Conductive Coupling of Split Ring Resonators: A Path to THz Metamaterials with Ultrasharp Resonances. Phys. Rev. Lett. 112, 183903 (2014). 24856698 10.1103/PhysRevLett.112.183903

[b19] Habteyes T. G., Dhuey S., Cabrini S., Schuck P. J. & Leone S. R. Theta-Shaped Plasmonic Nanostructures: Bringing “Dark” Multipole Plasmon Resonances into Action via Conductive Coupling. Nano Lett. 11, 1819–1825 (2011). 21425843 10.1021/nl200585b

[b20] Panaro S., De Angelis F. & Toma A. Dark and bright mode hybridization: From electric to magnetic Fano resonances. Opt. Laser. Eng. 76, 64–69 (2016).

[b21] Verellen N. . Fano Resonances in Individual Coherent Plasmonic Nanocavities. Nano Lett. 9, 1663–1667 (2009). 19281254 10.1021/nl9001876

[b22] Shafiei F. . A subwavelength plasmonic metamolecule exhibiting magnetic-based optical Fano resonance. Nat. Nanotechnol. 8, 95–99 (2013). 23353675 10.1038/nnano.2012.249

[b23] Funston A. M., Novo C., Davis T. J. & Mulvaney P. Plasmon Coupling of Gold Nanorods at Short Distances and in Different Geometries. Nano Lett. 9, 1651–1658 (2009). 19271775 10.1021/nl900034v

[b24] Slaughter L. S., Wu Y., Willingham B. A., Nordlander P. & Link S. Effects of Symmetry Breaking and Conductive Contact on the Plasmon Coupling in Gold Nanorod Dimers. Acs Nano 4, 4657–4666 (2010). 20614909 10.1021/nn1011144

[b25] Cui A. J. . Directly patterned substrate-free plasmonic “nanograter” structures with unusual Fano resonances. Light-Sci. Appl. 4, e308 (2015).

[b26] Katsarakis N., Koschny T., Kafesaki M., Economou E. N. & Soukoulis C. M. Electric coupling to the magnetic resonance of split ring resonators. Appl. Phys. Lett. 84, 2943–2945 (2004).

[b27] Fedotov V. A., Rose M., Prosvirnin S. L., Papasimakis N. & Zheludev N. I. Sharp trapped-mode resonances in planar metamaterials with a broken structural symmetry. Phys. Rev. Lett. 99, 147401 (2007). 17930720 10.1103/PhysRevLett.99.147401

[b28] Chen W. T. . Optical magnetic response in three-dimensional metamaterial of upright plasmonic meta-molecules. Opt. Express 19, 12837–12842 (2011). 21716526 10.1364/OE.19.012837

[b29] Huang Y. W. . Design of plasmonic toroidal metamaterials at optical frequencies. Opt. Express 20, 1760–1768 (2012). 22274519 10.1364/OE.20.001760

[b30] Liu N. . Three-dimensional photonic metamaterials at optical frequencies. Nat. Mater. 7, 31–37 (2008). 18059275 10.1038/nmat2072

[b31] Ebbesen T. W., Lezec H. J., Ghaemi H. F., Thio T. & Wolff P. A. Extraordinary optical transmission through sub-wavelength hole arrays. Nature 391, 667–669 (1998).

[b32] Ruan Z. C. & Qiu M. Enhanced transmission through periodic arrays of subwavelength holes: The role of localized waveguide resonances. Phys. Rev. Lett. 96, 233901 (2006). 16803379 10.1103/PhysRevLett.96.233901

[b33] Liu H. T. & Lalanne P. Microscopic theory of the extraordinary optical transmission. Nature 452, 728–731 (2008). 18401405 10.1038/nature06762

[b34] Georgiou G., Tserkezis C., Schaafsma M. C., Aizpurua J. & Rivas J. G. Active loaded plasmonic antennas at terahertz frequencies: Optical control of their capacitive-inductive coupling. Phys. Rev. B 91, 125443 (2015).

[b35] Kildishev A. V., Boltasseva A. & Shalaev V. M. Planar Photonics with Metasurfaces. Science 339, 6125 (2013). 10.1126/science.1232009 23493714

[b36] Yu N. F. & Capasso F. Flat optics with designer metasurfaces. Nat. Mater. 13, 139–150 (2014). 24452357 10.1038/nmat3839

